# Integrated transcriptome and single-cell RNA sequencing identifies transient receptor potential channel-related biomarkers in ulcerative colitis

**DOI:** 10.3389/fmolb.2026.1902447

**Published:** 2026-10-02

**Authors:** Tiantian Bao, Tianru Xie, Jiang Chen, Yibo Cao, Jian Zhu, Pingliang Sun, Tao Yang

**Affiliations:** 1 Colorectal and Anal Surgery, The First Affiliated Hospital of Guizhou University of Traditional Chinese Medicine, Guiyang, China; 2 College of Clinical Medicine, Guangxi University of Traditional Chinese Medicine, Nanning, China; 3 Department of Gastroenterology, The Affiliated Jinyang Hospital of Guizhou Medical University, Guiyang, China

**Keywords:** B cells, IL-1β, PIK3R3, transient receptor potential channel, ulcerative colitis

## Abstract

**Background:**

The precise cause of ulcerative colitis (UC) is still unclear. While transient receptor potential (TRP) ion channels are increasingly linked to UC pathophysiology, the comprehensive roles of TRP channel-related genes are not well understood. This study aimed to systematically identify TRP channel-related biomarkers and develop novel diagnostic and therapeutic targets for UC.

**Methods:**

Transcriptomic data were retrieved from the Gene Expression Omnibus (GEO). Machine learning algorithms were applied to select signature genes from the overlap between differentially expressed genes (DEGs) and a curated set of 111 transient receptor potential (TRP) channel-related genes. A diagnostic nomogram incorporating signature genes was constructed, and its performance was assessed by receiver operating characteristic (ROC) curves, calibration plots, and decision curve analysis (DCA). The cellular landscape was characterized using single-cell RNA sequencing (scRNA-seq) combined with pseudotime trajectory inference and cell–cell communication analysis. Key results were further validated in a dextran sulfate sodium (DSS)-induced murine colitis model by flow cytometry, multiplex immunohistochemistry, western blotting, and quantitative real-time PCR.

**Results:**

Five TRP channel-related feature genes (IL-1β, PIK3R3, ADCY3, ADCY7, and TRPV1) were identified. In UC, IL-1β, PIK3R3, ADCY3, and ADCY7 were notably upregulated, while TRPV1 was markedly downregulated. The nomogram, utilizing these five feature genes, exhibited outstanding discriminatory capability (AUC = 0.993) and provided enhanced net clinical benefit. scRNA-seq analysis identified B and plasma cells as the most significantly expanded pathogenic population, exhibiting acquired bidirectional crosstalk with epithelial cells in UC. Pseudotime trajectory analysis revealed that IL-1β and PIK3R3 were mainly concentrated during the initial developmental phases of B and plasma cells. In the DSS-induced colitis model, there was a notable increase in B-cell infiltration, accompanied by significant upregulation of IL-1β and Pik3r3 at both protein and mRNA levels, with confirmed colocalization with Cd19^+^ B cells.

**Conclusion:**

This study identified five TRP channel-related biomarkers and created a high-performance diagnostic nomogram for UC. The study underscores the pivotal role of B and plasma cells, identifying IL-1β and PIK3R3 as early-activated genes in pathogenic B-cell responses, thus offering new diagnostic biomarkers and therapeutic targets for UC.

## 1 Introduction

Ulcerative colitis (UC) is a chronic, relapsing inflammatory bowel disease (IBD) with a rising global incidence and substantial disease burden. The worldwide prevalence of IBD is projected to exceed 1% by 2035 (Kaplan, 2025), with an estimated 3.83 million prevalent cases in 2021 (Yang et al., 2025). Incidence has stabilized in early industrialized regions, but is increasing markedly in newly industrialized countries (Hracs et al., 2025), particularly in Asia (Zheng et al., 2025). By 2032, the age-standardized prevalence of IBD in Japan is predicted to be comparable to that in the United States (Zhang et al., 2025). Acute severe UC (ASUC) has a 12-month mortality rate of approximately 1.01% (Dong et al., 2020). UC severely impairs quality of life and can lead to serious complications.

The pathogenesis of UC involves genetic predisposition (Tripathi et al., 2025), environmental factors, intestinal dysbiosis (Han et al., 2025), impaired intestinal mucosal barrier function (Selvakumar and Samsudin, 2025), and aberrant immune-inflammatory responses (Wang H et al., 2025), yet the exact mechanisms remain unclear. Recent studies suggest that various gastrointestinal receptors, particularly transient receptor potential (TRP) ion channels, play a significant role in the pathophysiology of UC. TRP channels are versatile ion channels that detect a variety of chemical and physical stimuli. These channels are widely distributed in the GI tract, where they exert pleiotropic effects, participating in somatic and visceral nociception as well as in the maintenance of GI physiological functions (Khalil et al., 2018). Activation of TRP channels can trigger neurogenic inflammation, an inflammatory response caused by the release of immunomodulatory neuropeptides, especially calcitonin gene-related peptide (CGRP) and substance P (SP), from unmyelinated afferent neurons (Engel et al., 2011). TRP channels are also present in diverse immune cells, where they mainly regulate crucial immune functions like cytokine release, cell migration, and phagocytosis (Dvornikova et al., 2025). Owing to their dual roles as multimodal sensors and immunomodulators, TRP channels are thought to be critically involved in visceral hypersensitivity and the immune-mediated pathogenesis of UC (Zielińska et al., 2015; Chen et al., 2020). TRPV1 gene expression is notably elevated in colonic tissues of patients with active UC compared to those in remission, with its levels closely linked to the age of disease onset and clinical activity (Toledo-Mauriño et al., 2018). TRPA1 gene deficiency worsens CD4^+^ T cell-mediated colitis by lifting the inhibition on TRPV1 channels (Bertin et al., 2017), while TRPM7 gene knockdown may offer protection against UC by inhibiting the NLRP3 inflammasome-mediated pyroptosis pathway (Peng et al., 2024). However, the exact role of TRP channels in UC, including identified molecular targets and clinically relevant insights, is not well understood. Many studies concentrate on individual molecules without directly linking them to clinical outcomes or developing a systematic prognostic model based on TRP channel-related genes (Dvornikova et al., 2025). Consequently, their regulatory networks remain largely unclarified. Investigating the molecular mechanisms of TRP channel-related genes is crucial for enhancing early detection, discovering new biomarkers, and identifying potential therapeutic targets in UC.

This study aims to systematically identify TRP channel-related genes in UC and develop a prognostic risk model. Utilizing integrated analyses such as differential expression analysis of TRP channel biomarkers, nomogram development and evaluation, machine learning-based feature selection, single-cell RNA sequencing (scRNA-seq), and molecular expression validation, we clarified the molecular roles of TRP channel pathways in UC pathogenesis and occurrence. Our findings hold promise for refining clinical management and guiding the development of novel therapies for UC, ultimately advancing precise diagnostics and personalized interventions to improve patient outcomes.

## 2 Materials and methods

### 2.1 Data collection

Transcriptomic data for the training cohort were obtained from the GEO dataset GSE87466 (platform GPL13158) (Chen et al., 2022), which contains colon tissue profiles from 87 UC patients and 21 healthy controls (HC). For independent validation, the GSE75214 dataset (platform GPL6244) (Qian et al., 2023) was downloaded from the same repository, comprising colonic transcriptomes of 74 UC and 11 HC samples. Single-cell resolution data were acquired from GSE214695 (platform GPL18573), including colon specimens from 6 UC patients and 6 HC. A compendium of 111 TRP channel-associated genes was assembled by integrating two gene sets: the Reactome TRP channel set, retrieved from the Molecular Signatures Database (MSigDB, http://www.gsea-msigdb.org), and the KEGG pathway “Inflammatory mediator regulation of TRP channels” (https://www.kegg.jp/entry/ko04750).

### 2.2 Differential expression analysis

Differential expression analysis was performed on the training cohort with the limma package (Ritchie et al., 2015) to identify differentially expressed genes (DEGs) between UC and HC samples, applying thresholds of adjusted *P* value <0.05 and |log2 fold change (FC)| > 0.5. The resulting DEGs were visualized using ggplot2 (v 3.4.4) in a volcano plot, where the top 10 up- and downregulated genes ranked by |log2FC| were annotated. A heatmap of these top DEGs was generated with the ComplexHeatmap package (v 2.14.0) (Gu et al., 2016). Candidate genes jointly related to TRP channels and UC pathogenesis were obtained by intersecting the DEGs with the 111 TRP channel-related genes via the “Venn Diagram” package (v 1.7.3). A protein–protein interaction (PPI) network for the candidate genes was then constructed through the STRING database (https://string-db.org/, species: *Homo sapiens*, confidence score ≥0.4), from which disconnected nodes were removed. The final PPI network was visualized with the circlize package (v 0.4.15).

### 2.3 Gene Ontology and pathway enrichment analyses

To explore the biological functions and signaling pathways implicated in the candidate genes, enrichment analyses were performed using the clusterProfiler package (v 4.10.1). Gene Ontology (GO) analysis was conducted to annotate genes across three categories: Biological Process (BP), Cellular Component (CC), and Molecular Function (MF). Concurrently, Kyoto Encyclopedia of Genes and Genomes (KEGG) pathway analysis was employed to identify enriched metabolic and signal transduction pathways. For both analyses, an adjusted *P* value below 0.05 was considered statistically significant. The resulting terms were ranked by ascending adjusted *P* value, and the five most significant terms from each category were selected for visualization.

### 2.4 Machine learning

To identify feature genes associated with TRP channel-related pathways in ulcerative colitis (UC), two machine learning methods were sequentially applied to the training cohort. First, Least Absolute Shrinkage and Selection Operator (LASSO) regression was performed using the glmnet package (v 4.1–4), with the optimal model determined by 5-fold cross-validation. Next, Support Vector Machine Recursive Feature Elimination (SVM-RFE) was implemented via the caret package (v 6.0–93), in which features were iteratively eliminated from the candidate gene set based on linear kernel SVM weights, and classification accuracy was evaluated through 5-fold cross-validation. The gene subset achieving the lowest error rate was retained. The intersection of genes selected by LASSO and SVM-RFE was defined as the final set of feature genes.

### 2.5 Validation of feature genes

To confirm the expression consistency of the feature genes across datasets, the Wilcoxon rank-sum test was applied separately within the training and validation cohorts. Genes displaying significant differential expression (*P* < 0.05) with concordant direction of change between UC and HC samples in both cohorts were designated as biomarkers. The diagnostic performance of these biomarkers was assessed through receiver operating characteristic (ROC) curve analysis, with an area under the curve (AUC) greater than 0.7 considered indicative of discriminatory potential. This analysis was implemented using the pROC package (v 1.18.0).

### 2.6 Nomogram construction

Using the expression levels of the biomarkers in the training cohort, a nomogram was constructed with the rms package (v 6.5.0) to estimate individual UC risk, where a total score derived from the biomarkers was used to compute the predicted probability of disease onset. Model performance was assessed through multiple complementary approaches: ROC curves and corresponding AUC values were generated using the pROC package (v 1.18.0); calibration plots, illustrating the agreement between predicted probabilities and observed UC rates, were produced with the rms package; and decision curve analysis (DCA) was carried out with the rmda package (v 1.6) to evaluate the net clinical benefit of the nomogram across a range of threshold probabilities.

### 2.7 Gene set enrichment analysis (GSEA)

To explore biological pathways associated with the identified biomarkers in UC, we first computed Spearman correlation coefficients between each biomarker and all other genes in the training cohort using the psych package (v 2.1.6). Genes were then ranked by their correlation values to generate a pre-ranked gene list. GSEA was subsequently performed with the clusterProfiler package (v 4.7.1.003), applying the hallmark gene set (h.all.v2024.1. Hs.symbols.gmt) from the Molecular Signatures Database (MSigDB) as the reference. Enriched pathways were considered significant at |normalized enrichment score (NES)| > 1 and adjusted *P* < 0.05.

### 2.8 Correlations between biomarkers and differential immune cells

Single-sample gene set enrichment analysis (ssGSEA) was performed using the GSVA package (v 1.46.0) to estimate the infiltration scores in the training cohort. The resulting enrichment profiles were visualized as a heatmap with the pheatmap package (v 1.0.12), and differential immune infiltration between UC and HC samples was assessed using Wilcoxon tests. Immune cell types with an adjusted *P* value below 0.05 were designated as differential immune cells. Associations between the identified biomarkers and these differential immune cells were subsequently evaluated by Spearman correlation analysis via the psych package (v 2.2.9), with significance defined as |correlation coefficient| > 0.3 and *P* < 0.05. Significant correlations were displayed in a correlation plot generated using the corrplot package (v 0.95).

### 2.9 scRNA-seq analysis

Single-cell RNA sequencing data from the GSE214695 dataset were processed and analyzed using the Seurat package (v5.0.1) (Luo et al., 2017). Cells were filtered according to uniform criteria: 200–2,000 detected genes per cell (nFeature_RNA), total UMI counts ≤10,000 (nCount_RNA), and mitochondrial transcript percentage <25%. The distributions of nCount_RNA, nFeature_RNA, and mitochondrial proportion were displayed before and after quality control. Following data normalization, the top 2,000 highly variable genes (HVGs) were selected via the variance-stabilizing transformation (vst) method, with the 10 most variable genes highlighted. These HVGs were subsequently used for principal component analysis (PCA) and downstream dimensionality reduction. Batch effects were corrected within Seurat, and statistically significant principal components were identified using the JackStraw test (*P* < 0.05) for subsequent analyses. Cell clustering was then conducted with the FindNeighbors and FindClusters functions (resolution = 0.4), and the resulting clusters were visualized by t-distributed stochastic neighbor embedding (tSNE). Marker genes for each cluster were identified using the FindAllMarkers function, and their expression patterns were depicted in bubble plots. Major cell populations were annotated according to known marker genes. To pinpoint key cell types contributing to UC pathology, the proportional distribution of each annotated cell type across UC and HC samples was calculated, and intergroup differences were assessed using the Chi-square test. Cell types exhibiting significant proportional differences between groups and displaying differential biomarker expression were defined as key cells.

### 2.10 Cell-cell communication

Cell–cell communication was analyzed using the CellChat package (v1.6.1) on both UC and HC samples from the GSE214695 dataset, and the resulting interaction numbers and strengths were visualized.

### 2.11 Pseudotemporal ordering of key cells

Pseudotime analysis was performed on the key cells using the monocle R package (v2.26.0). The DDRTree algorithm was applied to construct a single-cell trajectory, elucidating the developmental and differentiation paths of these cells. Expression dynamics of the biomarkers along the pseudotime continuum were further visualized via trajectory plots.

### 2.12 Preparation of experimental animals

The model using dextran sulfate sodium (DSS)-induced mice with UC was previously reported by our group (Liu et al., 2024). Specific pathogen-free (SPF), male C57BL/6 J mice (6 weeks, weight: 20 ± 2 g) were purchased from Xiamen Mogengel Biotechnology Co., Ltd., (Permission No. SCXK (Min) 2025-0003). Mice were housed under specific pathogen-free (SPF) conditions in a controlled environment with access to sterile food and water. All animal procedures were conducted in accordance with protocols reviewed and approved by the Animal Ethical and Experimental Committee of Guizhou University of Traditional Chinese Medicine (Approval ID: KT-2023561). Following a 1-week acclimatization period, animals were randomly assigned to either the control or the UC group (n = 10 per group). Colitis was induced by providing 3% (w/v) dextran sulfate sodium (DSS; MP Biomedicals, CA, United States) in sterile drinking water *ad libitum* for 7 consecutive days. During this period, general health status, body weight, stool consistency, and the presence of rectal bleeding were recorded daily. On day 8, all mice were euthanized under deep anesthesia via a two-step procedure. Anesthesia was induced and maintained with isoflurane (3%–5% in oxygen for induction in an induction chamber until loss of the pedal withdrawal reflex). After confirmation of deep anesthesia, cervical dislocation was performed as the physical method of euthanasia. All procedures complied with the institutional animal care and use guidelines. Then, colon length was measured, and colon tissues were collected for paraffin embedding or snap-frozen in liquid nitrogen for subsequent analyses. The disease activity index (DAI) was calculated as the composite score of weight loss, stool consistency, and occult blood.

### 2.13 Hematoxylin-eosin (HE) staining of colon tissue

Paraffin-embedded colon specimens were sectioned at 5 μm and stained with hematoxylin and eosin (H&E). Based on the H&E-stained sections, the degree of inflammatory cell infiltration was assessed using the tissue damage index (TDI).

### 2.14 Flow cytometry (FCM) analysis

Lamina propria mononuclear cells were isolated from colonic tissues. Briefly, colons were stripped of mesentery and Peyer’s patches, opened longitudinally, and washed in ice-cold PBS. Tissues were cut into approximately 0.5 cm pieces and incubated in pre-digestion medium (HBSS without Ca^2+^/Mg^2+^, 5 mM EDTA, 1 mM DTT, 2% FBS) for 20 min at 37 °C with shaking to remove epithelial cells. After washing, the remaining fragments were digested in RPMI 1640 with 3% FBS, collagenase IV (1 mg/mL) and DNase I (0.1 mg/mL) for 40–45 min at 37 °C under continuous shaking. The cell suspension was passed through a 70-μm strainer, and mononuclear cells were enriched by 40%/80% Percoll gradient centrifugation (800 × g, 20 min, room temperature, without brake). Interface cells were collected, washed twice, and resuspended in FACS buffer (PBS with 1% FBS, 2 mM EDTA). For staining, 1 × 10^6^ cells were first incubated with anti-CD16/CD32 (Fc block, clone 2.4G2) for 15 min at 4 °C, followed by surface staining with anti-CD19 (clone 1D3), anti-CD3 (clone 145-2C11) and a fixable viability dye for 30 min at 4 °C. After two washes, cells were resuspended in FACS buffer and acquired on a flow cytometer; data were analyzed with FlowJo software. B cells were defined as live, single-cell CD19^+^CD3^−^ events.

### 2.15 Real-time quantitative PCR (RT-qPCR)

Total RNA was isolated from colon tissues using TRIzol reagent (Nordic Bioscience, Beijing, China) and subsequently reverse-transcribed with the PrimeScript RT reagent Kit containing gDNA Eraser (TaKaRa, Japan) following the manufacturer’s protocol. Briefly, 1 μg of RNA was treated with gDNA Eraser in a 10 μL reaction at 42 °C for 2 min to remove genomic DNA. Then, a reverse transcription mixture (total 20 μL) was added, and the reaction was incubated at 37 °C for 15 min, followed by enzyme inactivation at 85 °C for 5 s. The resulting cDNA was stored at −20 °C. Quantitative real-time PCR (qPCR) was performed on a 7,500 Fast Real-Time PCR System (Applied Biosystems) using SYBR Green PCR Master Mix (Nordic Bioscience). Each reaction (20 μL) consisted of 10 μL SYBR master mix, 0.8 μL of each primer (10 μM), 2 μL cDNA, and 6.4 μL nuclease-free water. The cycling program included an initial denaturation at 95 °C for 30 s, followed by 40 cycles of 95 °C for 5 s and 60 °C for 30 s, with fluorescence acquisition at the annealing/extension step. Melt curve analysis was performed from 65 °C to 95 °C (0.5 °C increments, 5 s each) to verify amplification specificity. Relative mRNA expression of IL-1β and Pik3r3 (primer sequences listed in Table 1) was calculated by the 2^−ΔΔCT^ method with Gapdh as the internal reference.

**TABLE 1:** Primer sequences for RT-qPCR experiment.

Genes	Forward (5‘-3’)	Reverse (5‘-3’)
Il-1β	GCAACTGTTCCTGAACTCAACT	ATCTTTTGGGGTCCGTCAACT
Pik3r3	TACAATACGGTGTGGAGTATGGA	CTCCACCACAGAAGTAAACGTC
Gapdh	AGGTCGGTGTGAACGGATTTG	TGTAGACCATGTAGTTGAGGTCA

### 2.16 Multiplex immunohistochemistry (mIHC) staining

Paraffin sections were deparaffinized in xylene and rehydrated through a graded ethanol series. mIHC was performed using a three-color mIHC kit (AFIHC027, AiFang Biological, China) according to the manufacturer’s instructions. Antigen retrieval was carried out by microwave heating. After quenching endogenous peroxidase with 3% hydrogen peroxide for 15 min at room temperature, the sections were blocked with 10% goat serum for 15 min. Primary antibody A was applied and incubated overnight at 4 °C, followed by three PBST washes. The sections were then incubated with polymer-HRP anti-rabbit universal secondary antibody IgG (AFIHC001, AiFang Biological) for 30 min at room temperature, washed, and reacted with TYR fluorophore for 8 min. After additional PBST washes, the bound antibodies were stripped by microwave treatment. This staining cycle was repeated sequentially for the remaining primary antibodies: Cd19 (AFRM0354, AiFang Biological, 1:500), IL-1β (YM8498, Immunoway, 1:500), and Pik3r3 (27035-1-AP, Proteintech, 1:500). Following the final staining round, nuclei were counterstained with DAPI for 10 min in the dark, and sections were coverslipped with anti-fade mounting medium. Images were acquired on a fluorescence digital slide scanner (AF-KL-20-8, AiFang Biological) and analyzed using HALO Image Analysis Software (Indica Labs, v4.2.26399.152).

### 2.17 Western blotting (WB) analysis

Colonic tissues were homogenized in ice-cold RIPA lysis buffer containing protease inhibitors at a tissue-to-buffer ratio of 1:10 (w/v). After centrifugation at 10,000 × g for 10 min at 4 °C, the supernatants were collected and stored at −20 °C. Protein concentrations were measured using a bicinchoninic acid (BCA) assay kit (Solarbio, Beijing, China). For Western blot analysis, equal amounts of protein (50 μg) were denatured in SDS loading buffer, separated by SDS-PAGE (80 V for 25 min, then 120 V for 50 min), and transferred to PVDF membranes (Millipore, MA, United States) at 200 mA for 2 h. Membranes were blocked with blocking buffer (NCM Bio, P30500) for 10 min at room temperature and subsequently incubated overnight at 4 °C with primary antibodies against pro-IL-1β (Proteintech, 16806-1-AP, 1:1,000), IL-1β (Immunoway, YM8498, 1:1,000), and Pik3r3 (Proteintech, 27035-1-AP, 1:1,000). After five washes with TBST (5 min each), the membranes were incubated with HRP-conjugated goat anti-rabbit secondary antibody (ZSGBBIO, ZB-2301, 1:25,000) for 1 h at room temperature with gentle agitation. Immunoreactive bands were visualized using a Bio-Rad detection system (Quantity One, United States) and quantified by densitometry. Gapdh (Proteintech, 60004-1-Ig, 1:10,000) served as the internal loading control for normalization.

## 3 Results

### 3.1 22 TRP channel-related genes were identified in the training cohort

From the training cohort, a total of 3,247 DEGs were identified, of which 1,948 were upregulated and 1,299 were downregulated in UC relative to HC (Figure 1A; Supplementary Table S1). Intersecting these DEGs with the set of 111 TRP channel-related genes yielded 22 candidate genes (Figure 1B; Supplementary Table S2). PPI network analysis revealed 45 co-expression interactions among these candidates (Figure 1C), implying their potential cooperative involvement in relevant regulatory processes. GO enrichment analysis assigned the 22 candidate genes to 735 terms, encompassing 653 BP, 22 CC, and 60 MF terms (Figure 1D; Supplementary Table S3). KEGG pathway analysis identified 148 enriched pathways, among which “Inflammatory mediator regulation of TRP channels” was prominently enriched (Figure 1D; Supplementary Table S4).

**FIGURE 1:**
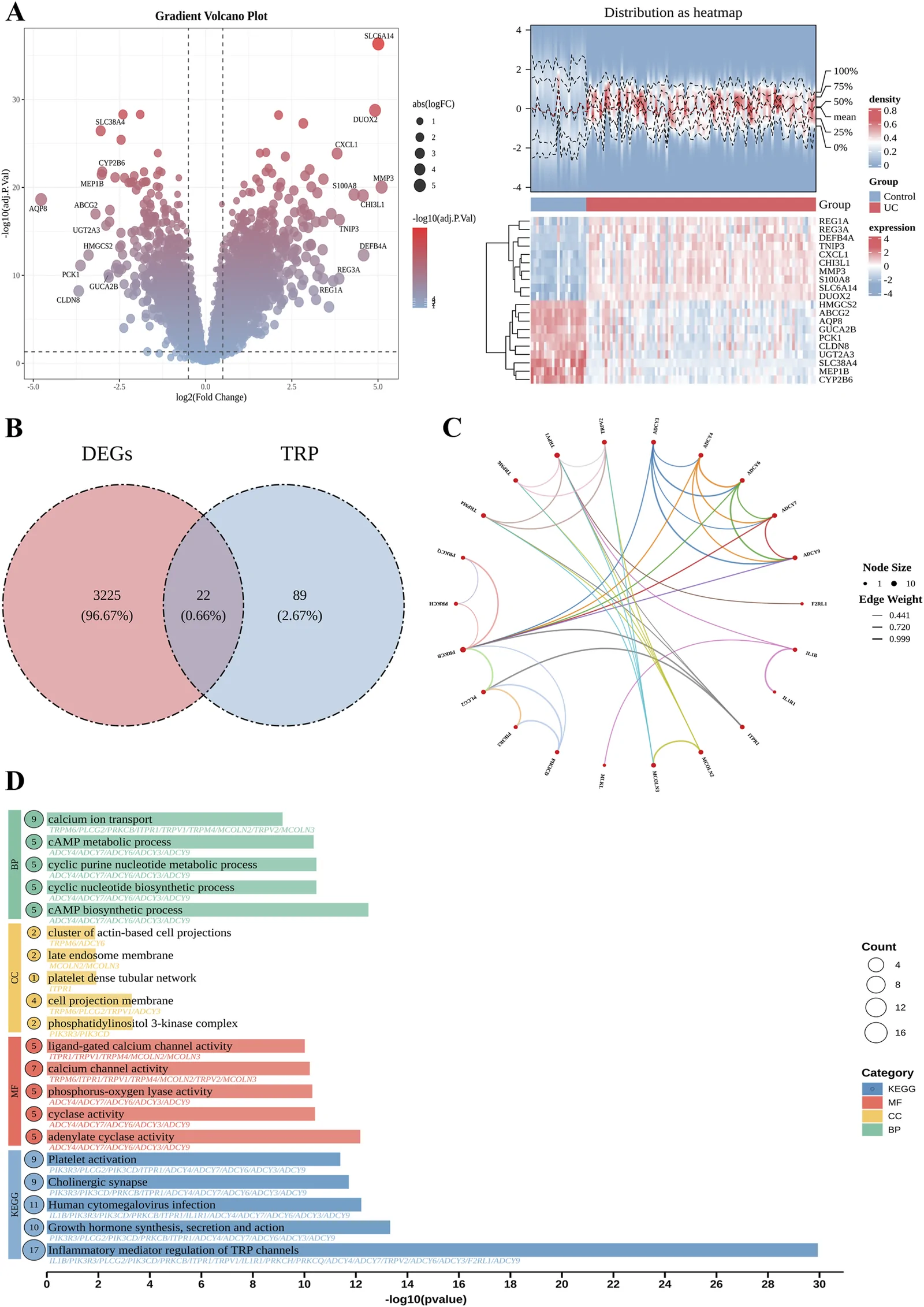
Identification of 22 candidate genes associated with TRP channel in UC. **(A)** Volcano plot of DEGs between UC and HC groups. Red and blue dots represent significantly upregulated and downregulated genes respectively. The top 10 up- and downregulated genes are labeled; **(B)** Venn diagram showing the intersection of 3,247 DEGs and 147 TRP channel-related genes, yielding 22 candidate genes; **(C)** PPI network of the 22 candidate genes; **(D)** GO and KEGG pathway enrichment analysis of the 22 candidate genes. The top 5 enriched pathways were presented.

### 3.2 5 TRP channel-related genes were identified as the feature genes

LASSO regression was performed to retain six genes (IL-1β, PIK3R3, ADCY3, ADCY7, TRPV1, ADCY4) at a lambda value of 0.01679854 (Figure 2A) from the 22 candidate genes. SVM_RFE algorithm refined this set to 19 robust features, including IL-1β, PIK3R3, TRPM6, MLKL, ITPR1, TRPV2, ADCY3, PRKCB, PRKCH, IL1R1, ADCY7, PLCG2, PIK3CD, TRPV1, PRKCQ, MCOLN2, ADCY9, TRPM4, MCOLN3 (Figure 2B). The intersection of these two algorithmic outputs yielded 5 shared feature genes (IL-1β, PIK3R3, ADCY3, ADCY7, TRPV1, Figure 2C). To further assess the discriminatory potential of those 5 feature genes, ROC analysis was performed. IL-1βachieved an AUC of 0.966 (95% CI: 0.920–0.998, Figure 2D), AUC for PIK3R3 was 0.956 (95% CI: 0.920–0.985, Figure 2E), ADCY3 achieved an AUC of 0.893 (95% CI: 0.805–0.961, Figure 2F), AUC for ADCY7 was 0.877 (95% CI: 0.791–0.949, Figure 2G) and for TRPV1 was 0.845 (95% CI: 0.791–0.949, Figure 2G) for ADCY7 was 0.877 (95% CI: 0.717–0.943, Figure 2H), all exceeding the threshold of 0.8 indicative of strong diagnostic utility for UC diagnosis.

**FIGURE 2:**
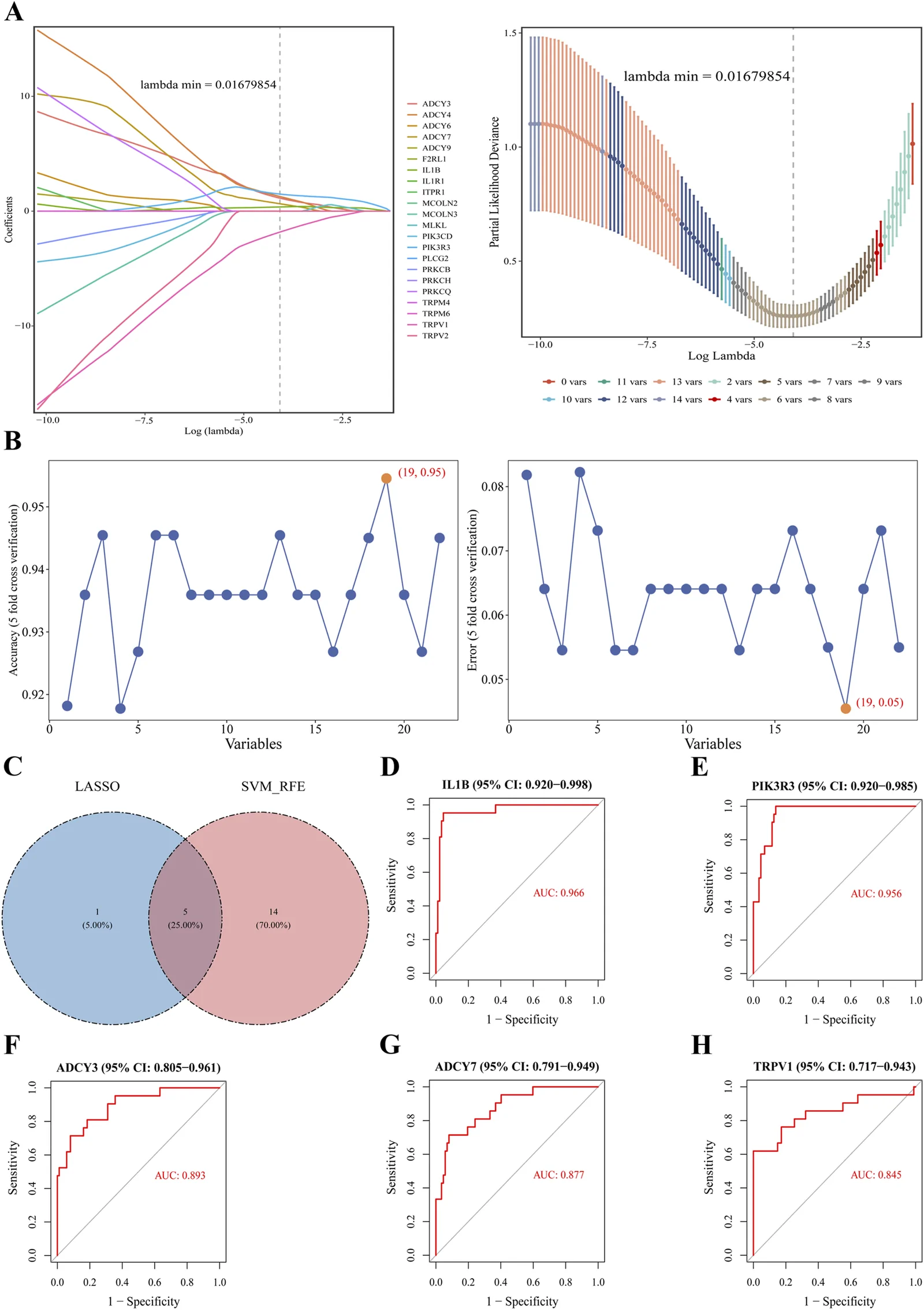
Identification of TRP channel-related feature genes in UC. **(A)** LASSO regression analysis with 5-fold cross-validation. The vertical dashed line indicates the optimal penalty parameter (λ). **(B)** Feature selection performed by the SVM-RFE algorithm; **(C)** Venn diagram showing the overlap between LASSO- and SVM-RFE-selected genes, resulting in five shared feature genes; **(D)** ROC analysis of IL-1β; **(E)** ROC analysis of PIK3R3; **(F)** ROC analysis of ADCY3; **(G)** ROC analysis of ADCY7; **(H)** ROC analysis of TRPV1.

### 3.3 Construction and validation of a nomogram based on the 5 TRP channel-related feature genes

In the training cohort (GSE87466), four feature genes, IL-1β, PIK3R3, ADCY3 and ADCY7, were markedly upregulated in UC relative to HC, while TRPV1 was significantly downregulated (^***^ *P* < 0.001, Figure 3A). These expression patterns were consistently reproduced in the independent validation cohort (GSE75214), with the four genes showing elevated expression (^**^ *P* < 0.01, ^***^ *P* < 0.001) and TRPV1 remaining suppressed (^***^ *P* < 0.001, Figure 3B). ROC analysis in the validation set yielded AUC values exceeding 0.9 for IL-1β, PIK3R3, ADCY7 and TRPV1, whereas ADCY3 achieved an AUC of 0.747 (95% CI: 0.571–0.903) (Figure 3C). A diagnostic nomogram integrating all five feature genes was constructed to estimate individual UC risk, and a total score of 227 points corresponded to a high probability of disease (Figure 3D). Calibration assessment, supported by a non-significant Hosmer–Lemeshow test (*P* = 0.357), indicated strong agreement between predicted and observed outcomes (Figure 3E). DCA demonstrated that the nomogram provided greater net benefit than both the “treat all” and “treat none” strategies across a broad range of threshold probabilities (Figure 3F). The nomogram exhibited excellent discriminatory performance, with an AUC of 0.993 for predicting UC occurrence (Figure 3G). Meanwhile, results from the independent validation cohort (GSE75214) further demonstrated that the five-gene nomogram model retained good discrimination, calibration, and potential clinical utility (Supplementary Figure S1).

**FIGURE 3:**
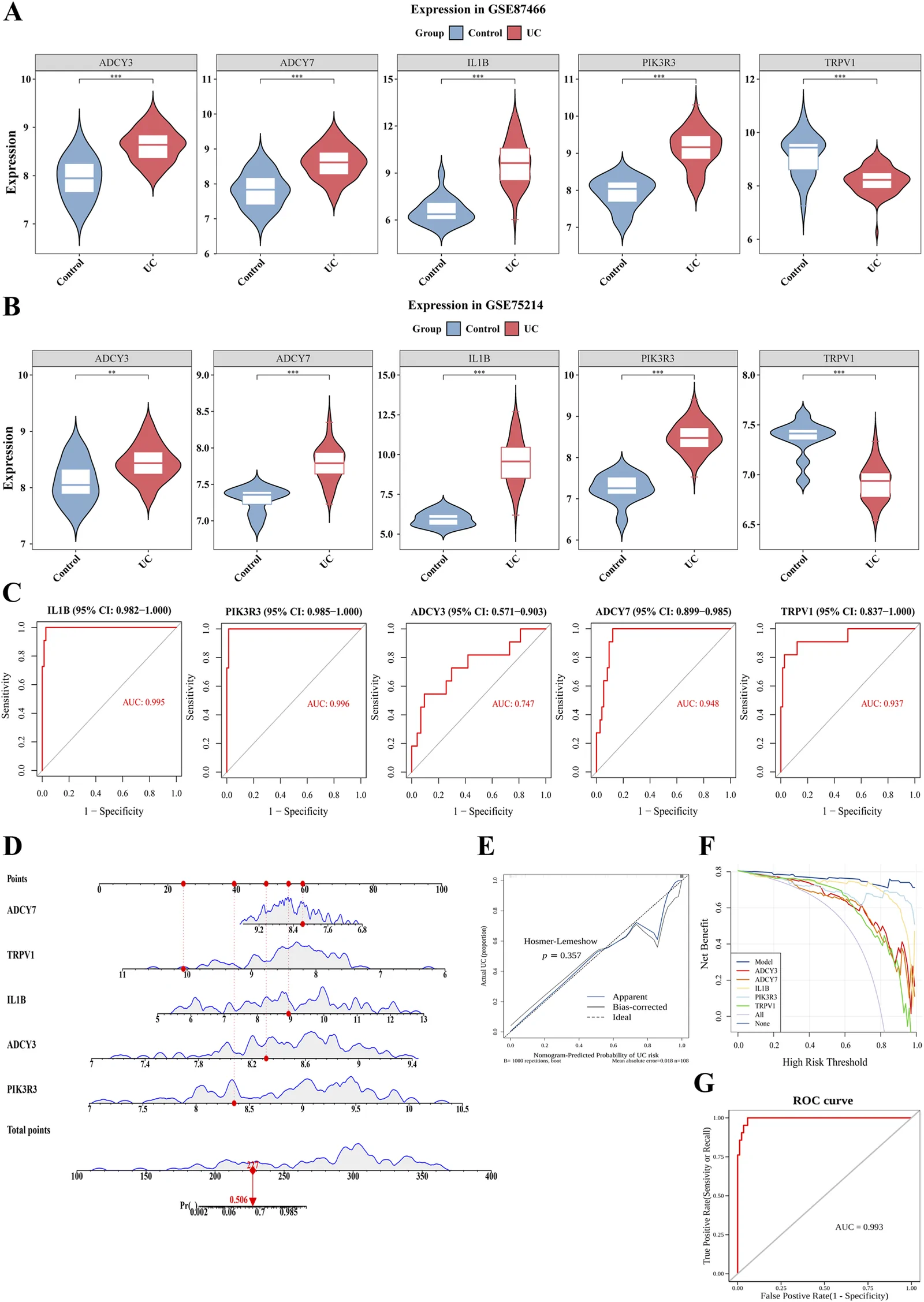
Nomogram construction and validation based on the expression of the 5 feature genes. **(A)** Violin plot of ADCY3, ADCY7, IL-1β, PIK3R3 and TRPV1 expression in the training cohort (GSE87466); **(B)** Violin plot of ADCY3, ADCY7, IL-1β, PIK3R3 and TRPV1 expression in the validation cohort (GSE75214); **(C)** ROC analysis of IL-1β, PIK3R3, ADCY3, ADCY7 and TRPV1 in the validation cohort (GSE75214); **(D)** Nomogram based on the expression of IL-1β, PIK3R3, ADCY3, ADCY7 and TRPV1; **(E)** Calibration curves for the predicted probability; **(F)** DCA of nomogram for UC; **(G)** ROC curve analysis of nomogram for UC probability.

### 3.4 GSEA of 5 TRP channel-related feature genes

To elucidate the biological pathways associated with the 5 TRP channel-related feature genes, GSEA was systematically performed. IL-1β was enriched in 93 pathways (Figure 4A; Supplementary Table S5), PIK3R3 in 93 pathways (Figure 4B; Supplementary Table S6), ADCY3 in 100 pathways (Figure 4C; Supplementary Table S7), ADCY7 in 93 pathways (Figure 4D; Supplementary Table S8), and TRPV1 in 75 pathways (Figure 4E; Supplementary Table S9), with 50 pathways shared among them (Supplementary Figure S2; Supplementary Table S10). These shared pathways included cell adhesion molecules, chemokine signaling, cytokine–cytokine receptor interaction, JAK-STAT signaling, B cell receptor signaling, PPAR signaling, and Toll-like receptor signaling, all of which are known to contribute to UC pathogenesis and progression. Correlation analysis further indicated that elevated expression of IL-1β, PIK3R3, ADCY3, and ADCY7 was significantly positively correlated with the upregulation of the cytokine–cytokine receptor interaction, JAK-STAT, and B cell receptor signaling pathways (Figure 4F), implying that these four genes may function as pathogenic drivers. In contrast, TRPV1 expression showed a significant positive correlation with the downregulation of pathways such as PPAR signaling (Figure 4F), suggesting a protective role. Collectively, these results highlight the distinct yet complementary functional contributions of the five TRP channel-related feature genes to the development and progression of UC.

**FIGURE 4:**
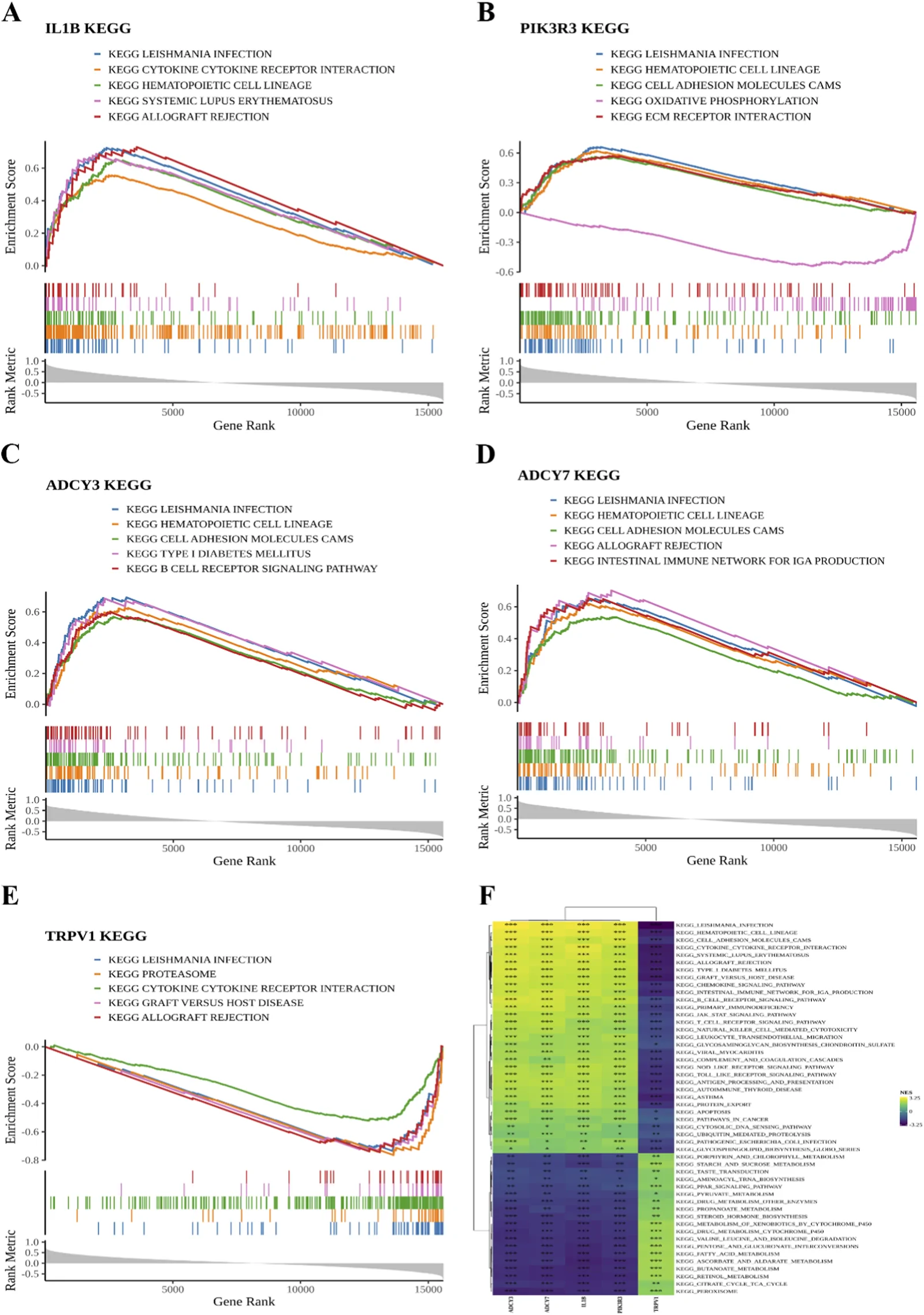
Functional pathway enrichment analysis of 5 TRP channel-related feature genes. **(A)** GSEA of IL-1β; **(B)** GSEA of PIK3R3; **(C)** GSEA of ADCY3; **(D)** GSEA of ADCY7; **(E)** GSEA of TRPV1; **(F)** Hotmap of correlation between 5 TRP channel-related feature genes and 50 shared pathways.

### 3.5 B and plasma cells were identified as key cell clusters in UC

To decipher the cellular atlas of UC, we analyzed scRNA-seq data from GSE214695. Based on the elbow plot, the top 50 principal components (PCs) at which the variance stabilized were selected for downstream analyses (Figure 5A). All cells were subsequently grouped into 22 clusters (Figure 5B). From these 22 clusters, 7 cell types were identified: B and plasma cells, endothelial cells, T cells, stromal cells, macrophages, myeloid cells, and NK cells (Figure 5C). Using the clustering results, cell clusters were annotated based on marker gene expression. The marker genes of the seven identified cell types displayed highly specific expression, confirming the reliability of the annotation (Figure 5D; Table 2). The proportions of these seven cell types were then compared (Figure 5E). Notably, the proportions of B and plasma cells and macrophages were markedly increased in UC, whereas the proportions of endothelial cells and NK cells were significantly decreased (Figure 5F). At the donor level, the proportion of B/plasma cells was overall higher in the UC group than in the control group (Figure 5G). B cells and plasma cells were further annotated as distinct cell populations (Figures 5H,I). Collectively, these results identified B cells and plasma cells as key cell clusters in UC.

**FIGURE 5:**
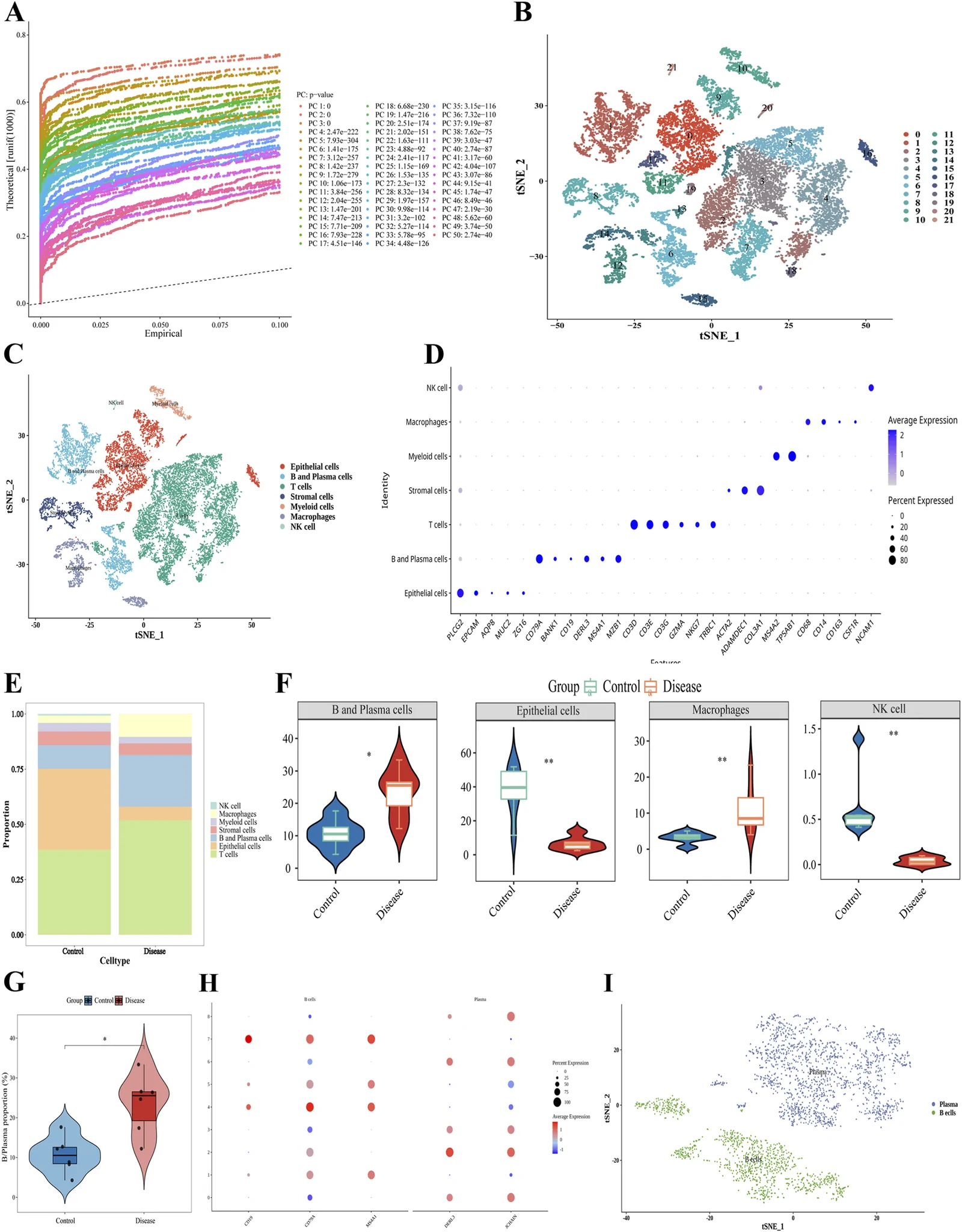
Identification and annotation of key cell types. **(A)** The top 50 PCs selection; **(B)** 22 clusters were identified by tSNE reduced dimensionality; **(C)** 7 cell types were identified by tSNE reduced dimensionality in UC and HC groups; **(D)** Dot plot of cell type marker genes; **(E)** The comparison of 7 cell types proportions; **(F)** Differential cell types between UC and HC groups; **(G)** The comparison of B/plasma cells in UC and HC groups at the donor level (^*^ *P* < 0.05); **(H)** Dot plot of B/plasma cells marker genes; **(I)** tSNE reduced dimensionality for B/plasma cells.

**TABLE 2:** Annotation of seven cell types.

Marker genes	Cluster	Annotation of cell types
PLCG2, EPCAM, AQP8, MUC2, ZG16	0, 9, 11, 16, 17, 19, 20	Epithelial cells
CD79A, BANK1, CD19, DERL3, MS4A1, MZB1	1, 6	B and plasma cells
CD3D, CD3E, CD3G, GZMA, NKG7, TRBC1	2, 3, 4, 5, 7, 13, 18	T cells
ACTA2, ADAMDEC1, COL3A1, PVALP	8	Stromal cells
MS4A2, TPSAB1, TPSAB2	10	Myeloid cells
CD68, CD14, CD163, CSF1R	12, 14, 15	Macrophages
NCAM1	21	NK cells

### 3.6 Identification of 5 TRP channel-related feature genes in B and plasma cells

We further analyzed the expression differences of five TRP channel-related feature genes across B and plasma cells. The results showed that ADCY3, ADCY7, TRPV1 were highly expressed in B cells (Figures 6A,B), IL-1β and PIK3R3 were highly expressed in plasma cells (Figures 6A,B). Between the UC and HC groups, IL-1β and PIK3R3 were significantly differentially expressed in plasma cells (Figure 6C), while ADCY3 was significantly differentially expressed in B cells (Figure 6C). However, the proportions of B cells and plasma cells did not differ significantly between the UC and HC groups (Figure 6D). These results suggest that B cells and plasma cells may serve as an integrated key cell population (Shapiro-Shelef and Calame, 2005). Cell–cell communication analysis based on interaction number networks and communication weight networks revealed no detectable interactions between B and plasma cells and epithelial cells in HC groups (Figure 6E). In contrast, bidirectional interactions between B and plasma cells and epithelial cells were observed in UC (Figure 6F). To further validate the gene expression patterns and the altered proportion of B and plasma cells, we employed a DSS-induced colitis mouse model. The UC group exhibited significant body weight loss (Figure 6G), and the DAI was notably worse compared to the control group (Figure 6H). Colon shortening was evident in DSS-induced colitis, confirming successful model establishment (Figure 6I). Furthermore, HE staining revealed that in the UC group, the typical mucosal architecture was destroyed, crypt structures were indistinct, goblet cells were lost, massive infiltration of inflammatory cells was present, and hemorrhagic spots appeared in the colonic mucosa (Figure 6J; Supplementary Figure S3). Flow cytometric analysis of colonic tissue demonstrated a significantly increased proportion of B/plasma cells in the UC group (Figure 6K; Supplementary Figure S4). Collectively, these results indicate that B/plasma cells represent a critical pathogenic cell population in UC.

**FIGURE 6:**
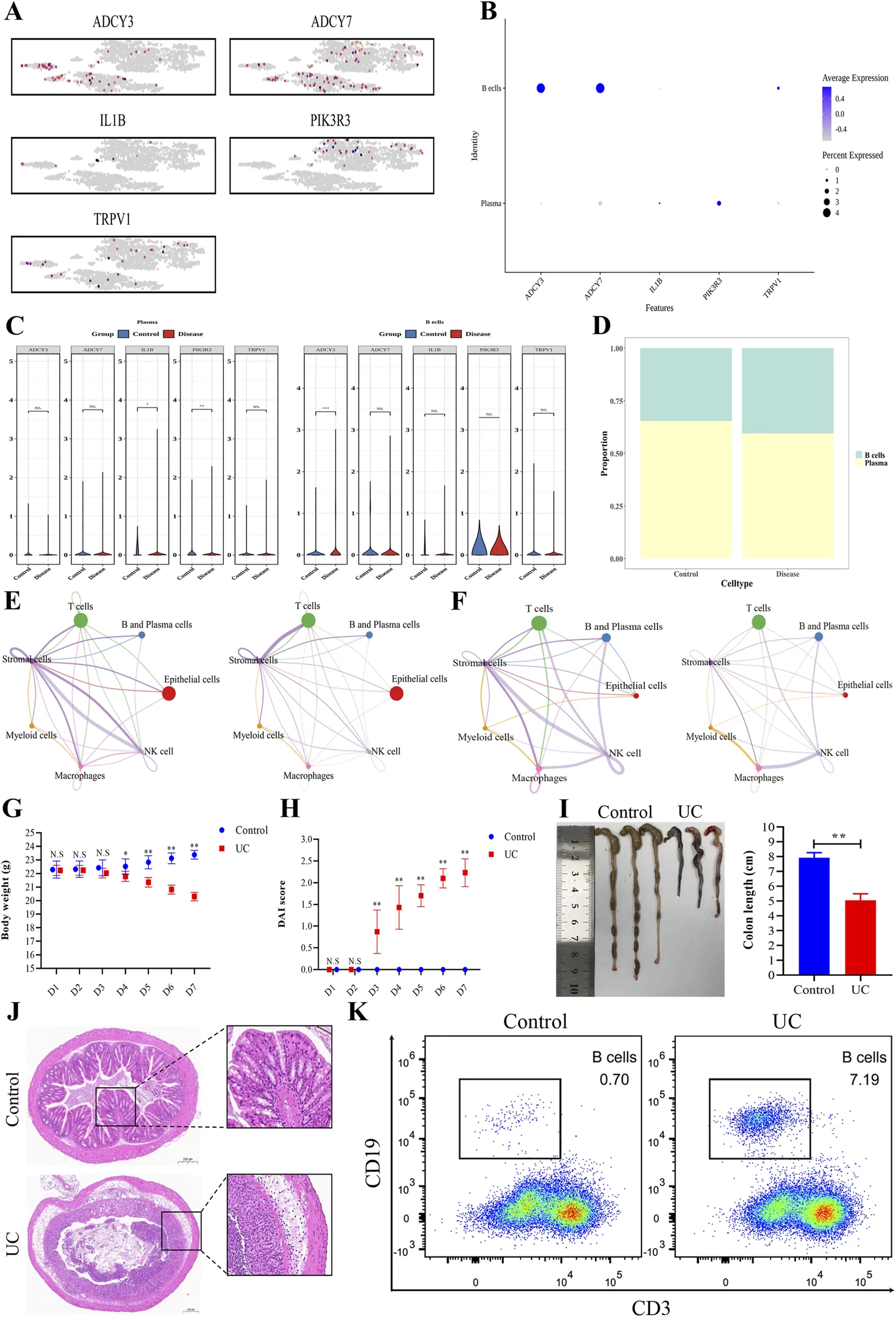
Differential cell clusters and cell–cell communication analysis in UC and HC samples. **(A)** Expression differences of five TRP channel-related feature genes across B and plasma cells; **(B)** Dot plot of five TRP channel-related feature genes in B and plasma cells; **(C)** Differential Expression of five TRP channel-related feature genes in B and plasma cells; **(D)** The comparison of B cells and plasma cells in UC and HC groups; **(E)** Interaction number networks and communication weight networks analysis in HC samples; **(F)** Interaction number networks and communication weight networks analysis in UC samples; **(G)** Body weight of each group mice (*n* = 10); **(H)** DAI values of each group mice (*n* = 10); **(I)** Colon length of each group mice; **(J)** HE staining of each group mice (*n* = 5). Scale bars = 200 μm; **(K)** Ratio of B cells in colonic tissues detected by FCM.

### 3.7 Pseudotime trajectory analysis of B and plasma cells

To delineate the developmental dynamics of B cells and plasma cells, we performed pseudotime trajectory analysis, with B cells set as the root state (root = 3) and plasma cells as the relatively late state. Based on the elbow plot, we selected the top 50 PCs at which variance stabilized (Figure 7A). All subcells were subsequently grouped into nine clusters by t-SNE dimensionality reduction (Figure 7B). We then colored B cells and plasma cells according to the B cells to plasma cells differentiation process. Darker blue indicated earlier differentiation, whereas the lightest blue denoted the most recently differentiated cells (Figure 7C), demonstrating that B cells and plasma cells gradually differentiate from left to right along the trajectory. Subsequently, we partitioned the developmental process into five states, with states 1 and 2 defined as early developmental stages and states 3 and 4 as late developmental stages. B cells and plasma cells were predominantly distributed in states 1 and 2 (Figure 7C). Notably, throughout the developmental trajectory, UC samples exhibited an increased distribution of cells at the late developmental stages (Figure 7C). We further examined the expression dynamics of the five TRP channel-related feature genes along the pseudotime trajectory of B cells and plasma cells. IL-1β and PIK3R3 exhibited higher expression levels at the early stage than at the middle and late stages, whereas ADCY3, ADCY7, and TRPV1 all displayed elevated expression at the late stage (Figure 7D). Consistently, IL-1β and PIK3R3 expression was predominantly enriched in state 1 and 2 (Figure 7E), suggesting that these two genes may be involved in the development of B cells into plasma cells.

**FIGURE 7:**
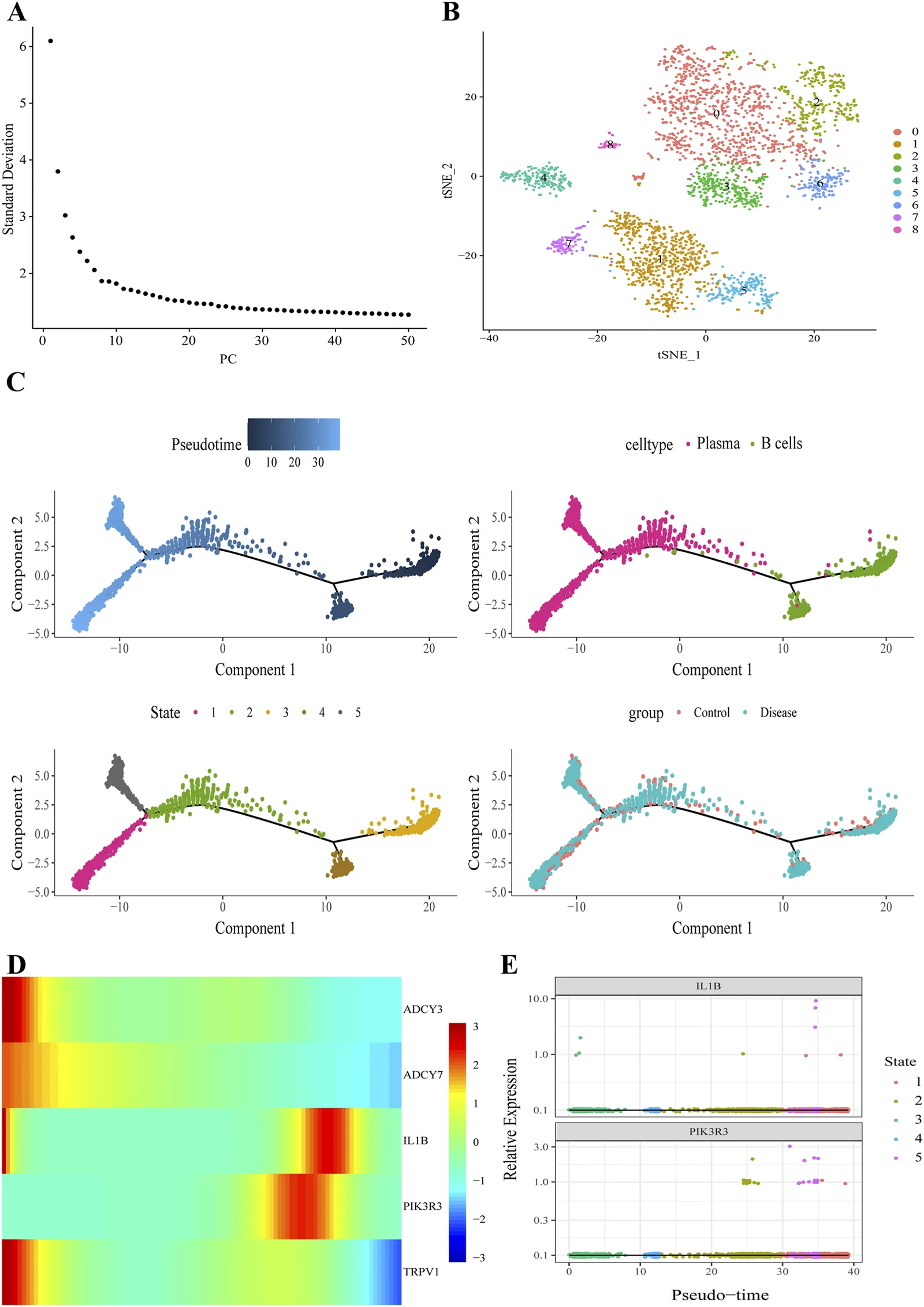
Pseudotime trajectory analysis and the expression dynamics of the five TRP channel-related feature genes in B and Plasma cells. **(A)** The top 50 PCs selection of B and Plasma cells; **(B)** 9 cell clusters were identified by tSNE reduced dimensionality in B and Plasma cells; **(C)** Pseudotime trajectory analysis from timing differences in cell differentiation, different cell clusters, developmental stages and different samples (HC and UC); **(D)** The expression dynamics of the five TRP channel-related feature genes along the pseudotime trajectory of B and plasma cells; **(E)** IL-1β and PIK3R3 expression in 5 developmental stages.

### 3.8 IL-1β and PIK3R3 were highly expressed in UC mouse model

In the training cohort, ssGSEA analysis revealed that IL-1β expression was positively correlated with both immature B cells and activated B cells (Figure 8A). Similarly, PIK3R3 expression showed a positive correlation with immature and activated B cells (Figure 8B). These results further corroborate the association of IL-1β and PIK3R3 expression with B cell infiltration in UC. mIHC co-localization analysis demonstrated that in the colonic tissues of mice in UC group, the positive rate of B cells (Cd19^+^) was markedly increased, and the positive rates of IL-1β and Pik3r3 were also significantly elevated (Figure 8C; Supplementary Figure S5). WB analysis showed that the protein expression levels of pro-IL-1β, IL-1β, and Pik3r3 were significantly upregulated in the colonic tissues of the UC group (Figure 8D). Moreover, the mRNA expression levels of IL-1β and Pik3r3 were significantly increased in the UC group compared to controls (Figure 8E). Collectively, these data demonstrate that IL-1β and PIK3R3 serve as consistent and reliable biomarkers for UC pathogenesis and diagnosis.

**FIGURE 8:**
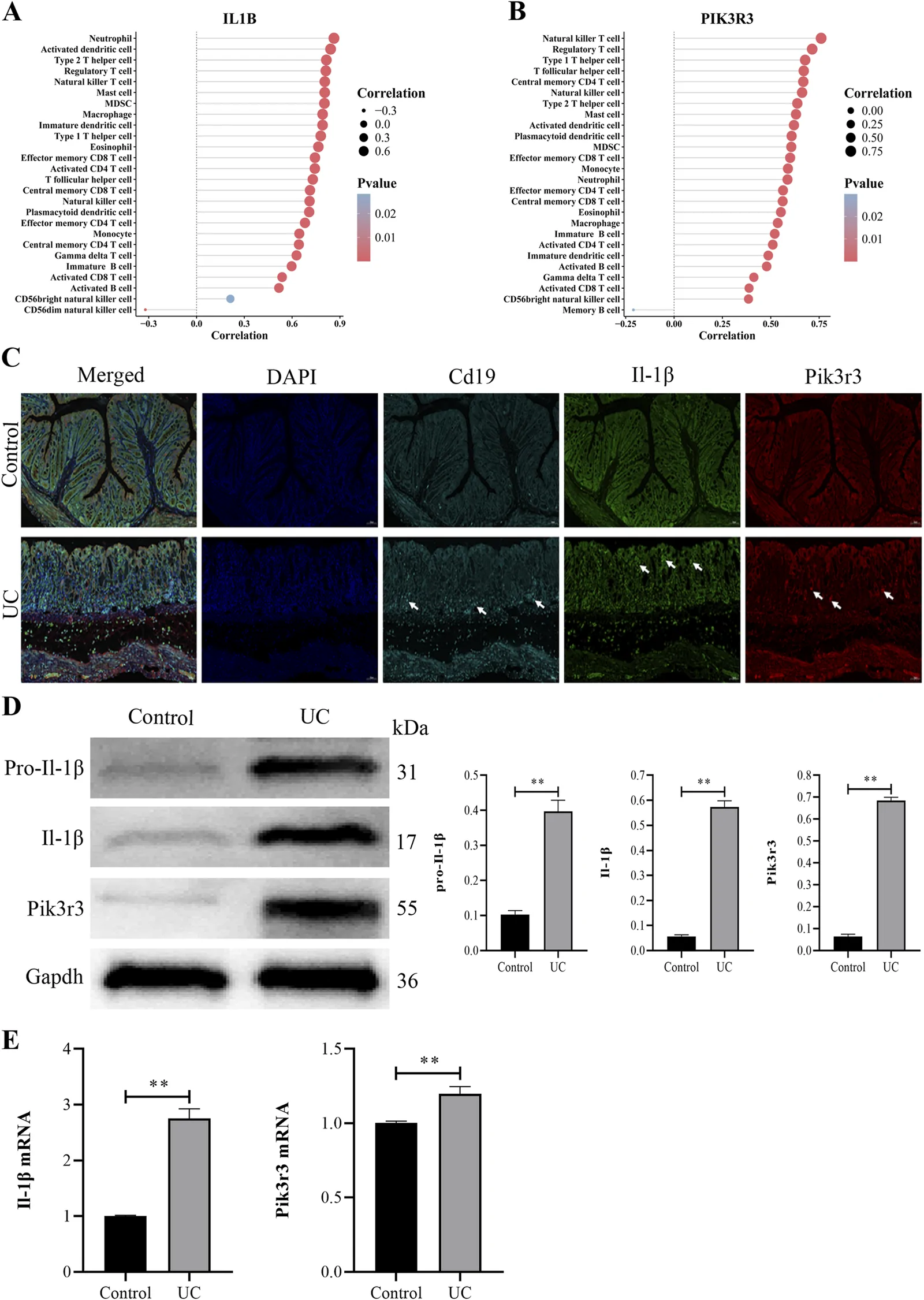
Validation of IL-1β and PIK3R3 expression in UC mouse model. **(A)** ssGSEA of IL-1β expression and immune-infiltrating cells; **(B)** ssGSEA of and PIK3R3 expression and immune-infiltrating cells; **(C)** mIHC images of the colonic tissues of mice between the UC and control groups; **(D)** Protein expression levels of pro-IL-1β, IL-1β, and Pik3r3 in the UC and control groups (^**^ *P* < 0.01); **(E)** Relative mRNA expression levels of IL-1β, and Pik3r3 in the UC and control groups (^**^ *P* < 0.01).

## 4 Discussion

UC is widely recognized as a multifactorial intestinal immune-inflammatory disease, yet its exact pathogenesis remains incompletely elucidated. In recent years, TRP ion channels have been increasingly recognized as potential key players in the pathophysiology of UC. In the present study, through bioinformatics analyses combined with experimental validation, we have preliminarily revealed the potential molecular roles of TRP channel-related genes in UC. We identified five TRP channel-related feature genes (IL-1β, PIK3R3, ADCY3, ADCY7, and TRPV1) and constructed a diagnostic nomogram with excellent discriminatory ability (AUC = 0.993) and clinical net benefit. Using scRNA-seq analysis and functional experiments, we found that B and plasma cells represent key pathogenic cell populations, and that IL-1β and PIK3R3 are highly expressed along the early developmental trajectory of these cells. Similarly, IL-1β and PIK3R3 expression showed a positive correlation with immature and activated B cells. These findings provide promising diagnostic tools, markers and potential therapeutic targets within the TRP channel-related molecular network in UC.

The five TRP channel-related feature genes harbor distinct biological functions and participate in the immunopathogenesis of UC. IL-1β encodes interleukin-1β (IL-1β), a core regulatory gene of the innate immune system and a major pro-inflammatory cytokine, and its upregulation in UC is well recognized. In the present study, ROC analysis showed that IL-1β achieved an AUC of 0.966 (95% CI: 0.920–0.998) in the training cohort and 0.995 (95% CI: 0.982–1.000) in the validation cohort, indicating excellent independent diagnostic performance (AUC > 0.9). IL-1β binds to the IL-1 receptor (IL-1R) and activates downstream NF-κB and MAPK signaling pathways, regulating the expression of multiple target genes and driving key inflammatory processes such as fever, the acute-phase response, and immune cell recruitment and activation (Hassaan et al., 2026). Thus, it is closely associated with various chronic inflammatory diseases, including UC (Zhao et al., 2025). Previous studies demonstrated that IL-1β upregulates myosin light chain kinase (MLCK) expression via NF-κB signaling, leading to the phosphorylation of intestinal tight junction proteins and increased intestinal epithelial barrier permeability, confirming that IL-1β is not merely an inflammatory mediator but a critical factor that directly disrupts intestinal barrier function (Al-Sadi et al., 2012). Shouval DS, et al. further established that IL-1β acts as a core driver of severe intestinal inflammation in patients with IL-10 receptor deficiency and in corresponding animal models. They found that loss of IL-10 signaling dysregulates the intestinal mucosal immune system, resulting in excessive and sustained production of IL-1β. This pro-inflammatory cytokine then activates downstream cascades, including NF-κB, recruiting and activating large numbers of neutrophils and macrophages that infiltrate the intestinal tissue, causing severe mucosal damage and ulceration. Moreover, by using IL-1β-blocking antibodies or genetic knockout approaches, they demonstrated that specific inhibition of IL-1β significantly attenuates intestinal pathological inflammation, reduces disease activity, and improves histological scores (Shouval et al., 2016). These findings not only reveal a critical pathological role for IL-1β in refractory intestinal inflammation but also provide experimental evidence and theoretical support for the clinical development of biologic agents targeting IL-1β for UC treatment. PIK3R3 (phosphoinositide-3-kinase regulatory subunit 3) is a key regulatory gene of the PI3K/AKT signaling pathway. It encodes the p55γ (also known as p55PIK) regulatory subunit of PI3K, which, together with the catalytic subunit p110, forms the class I PI3K heterodimer and plays a central role in cell growth, proliferation, survival, metabolism, and immune regulation. A study reported that PIK3R3 is significantly overexpressed in the colonic tissues of patients with IBD, which is consistent with our findings, and that it negatively regulates the expression of the tight junction protein ZO-1 through activation of the NF-κB signaling pathway, leading to impaired intestinal epithelial barrier function and increased intestinal permeability (Ibrahim et al., 2020). *In vitro* experiments demonstrated that PIK3R3 overexpression reduced the transepithelial electrical resistance (TEER) of Caco-2 cell monolayers, whereas PIK3R3 knockdown restored tight junction integrity. More importantly, treatment with TAT-N15, a specific PIK3R3 inhibitor, significantly improved intestinal permeability and mucosal barrier function in a DSS-induced colitis mouse model, indicating that PIK3R3 represents a potential therapeutic target in UC(29). Furthermore, Cheng C, et al. identified PIK3R3 as one of the specific hub genes for UC and Crohn’s disease (CD), which, together with GNG11, GNB4, AGT, and CCR7, constitutes a disease-specific molecular marker panel. The expression level of PIK3R3 can effectively distinguish UC from CD, providing a novel molecular basis for precise subtyping and diagnosis of IBD (Cheng et al., 2019).

ADCY3 and ADCY7 encode adenylyl cyclase isoforms that catalyze the conversion of ATP to cyclic AMP (cAMP). As a second messenger, cAMP possesses pleiotropic immunomodulatory properties. However, the pronounced upregulation of these cyclases observed in UC suggests dysregulated cAMP signaling that may contribute to immune cell activation and mucosal injury. In this study, GSEA confirmed that the expression of ADCY3 and ADCY7 was significantly and positively correlated with the upregulation of pro-inflammatory pathways, including cytokine–cytokine receptor interaction, the JAK-STAT signaling pathway, and the B cell receptor signaling pathway. Ren D, et al. identified ADCY3 as one of three core susceptibility genes for UC and found that it is specifically and highly expressed in colonic B cells rather than in epithelial cells, suggesting its involvement in UC pathogenesis through the regulation of B cell-mediated adaptive immune responses (Ren et al., 2025). Another study also identified ADCY3 as a core diagnostic biomarker in UC and provided preliminary evidence that ADCY3 represents a potentially druggable target for UC (Chen et al., 2024). Luo Y, et al. reported that ADCY7 is one of the strongest genetic susceptibility genes for UC (second only to the HLA region) and discovered that the low-frequency missense variant p. Asp439Glu (rs78534766, MAF = 0.6%) confers a 2.19-fold increased risk of UC(33). This variant results in loss of ADCY7 function, reduced cAMP production, and consequently excessive inflammatory responses. In contrast, TRPV1 was consistently downregulated in both the training and validation cohorts in the present study. TRPV1 is a key gene encoding a non-selective cation channel located on the short arm of human chromosome 17 (17p13.2). As a member of the TRP channel superfamily, TRPV1 plays a central role in nociception, inflammatory responses, and visceral sensory regulation (Yang and Duan, 2026; Zalecki et al., 2026). Previous studies demonstrated that TRPV1 knockout mice exhibited more severe colonic inflammation and tissue damage, suggesting that TRPV1 may exert protective effects by modulating neurogenic inflammation and intestinal immune homeostasis (Massa et al., 2006). This model-dependent difference reflects the complex bidirectional regulatory role of TRPV1 in intestinal inflammation, which varies according to the type, severity, and genetic background of the inflammatory condition.

In our scRNA-seq analysis, B and plasma cells were identified as the most prominently expanded pathogenic cell populations. Accumulating evidence has repositioned B cells from passive bystanders to active drivers in UC pathogenesis (Li et al., 2026; Fung et al., 2026; Chen et al., 2025). Beyond their canonical antibody-producing function, pathogenic B cells and antibody-secreting plasma cells accumulate extensively in the inflamed colonic mucosa, where they contribute to intestinal tissue injury through multiple mechanisms, including the secretion of pro-inflammatory cytokines such as IL-6, IL-35 and TNF-α, presentation of autoantigens to pathogenic T cells, and production of autoreactive antibodies that cross-react with commensal microbial antigens (Li et al., 2026; Wang J et al., 2025). Our cell–cell communication analysis further revealed a disease-specific pathological feature: while no detectable interactions between B and plasma cells and epithelial cells were observed in healthy tissues, robust bidirectional crosstalk emerged in UC samples. This acquired intercellular dialogue likely constitutes a self-amplifying inflammatory loop, wherein epithelium-derived signals promote B-cell activation and, in turn, B-cell-derived factors exacerbate epithelial injury and mucosal barrier disruption.

To clarify how TRP channel-related genes participate in this pathogenic B-cell response, we performed pseudotime trajectory analysis. The results showed that IL-1β and PIK3R3 were predominantly enriched at the early stages of B and plasma cell development. This expression pattern suggests that these two genes are not merely bystanders but are likely involved in the initial priming, survival, or maturation of pathogenic B cells in UC. IL-1β potently promotes B-cell proliferation and differentiation into antibody-secreting cells, enhances antigen presentation, and synergizes with IL-6 to drive plasma cell survival (Dinarello, 2018; Fillatreau, 2019). Therefore, its upregulation at the early B-cell trajectory implies that, in the inflamed colon, IL-1β may act as an autocrine or paracrine facilitator of early B-cell activation. PIK3R3 encodes the p55γ regulatory subunit of PI3K and likewise occupies a critical position in B-cell biology. The PI3K/AKT signaling pathway governs virtually every stage of B-cell development and function, from pre-B-cell expansion to germinal center formation and class-switch recombination (Safaroghli-Azar et al., 2023; Abrol and Deenick, 2026). Overexpression of PIK3R3 in early B-lineage cells would be predicted to potentiate PI3K pathway activity, lower the threshold for B-cell activation (Donahue and Fruman, 2004), and favor the survival of autoreactive clones, thereby sustaining the chronic inflammatory milieu in UC. We subsequently validated these findings in a DSS-induced colitis mouse model, which confirmed a significant increase in B-cell infiltration, upregulated protein and mRNA expression levels of both IL-1β and PIK3R3, and their colocalization with Cd19^+^ B cells in colonic tissues. Collectively, these observations establish IL-1β and PIK3R3 as biomarkers intimately linked to the B-cell-driven immune response in UC and provide experimental evidence for therapeutically targeting pathogenic B-cell responses to alleviate UC.

However, several limitations of this study should be noted. First, the analysis was based on microarray and scRNA-seq data with a relatively modest sample size. Although an independent validation cohort was employed, the generalizability of the nomogram still requires confirmation through prospective multicenter studies. Second, although we validated the expression of IL-1β and PIK3R3 in a mouse colitis model, functional perturbation experiments (such as conditional knockout or pharmacological inhibition) were not performed to establish causal relationships between these TRP channel-related genes and UC phenotypes. Third, the limited number of samples in the scRNA-seq data analyzed may have hindered the identification of rare cell subsets associated with TRP channel-mediated inflammation. Fourth, the diagnostic model distinguishes UC from healthy controls but has not been evaluated for its ability to differentiate UC from other inflammatory bowel disease subtypes or infectious colitis. Finally, 5 feature genes are merely associated with TRP channel pathways rather than being TRP channel subunits or direct regulators. The regulatory interplay between these genes and TRP ion channel activity was inferred primarily at the transcriptomic level, and direct electrophysiological and biochemical studies are required to elucidate the precise molecular mechanisms.

In summary, this study identified five TRP channel-related feature genes and developed a high-performance diagnostic nomogram for UC. Our findings demonstrate the critical involvement of B and plasma cells and reveal that IL-1β and PIK3R3 are crucial genes in pathogenic B and plasma cells responses. These findings not only deepen the molecular understanding of TRP channel-related biomarkers in the pathogenesis of UC, but also lay a foundation for the development of diagnostic approaches and targeted therapeutic strategies. Future studies should focus on the functional validation of TRP channel-related genes and their translation into clinical practice.

## Data Availability

The datasets presented in this study can be found in online repositories. The names of the repository/repositories and accession number(s) can be found in the article/Supplementary Material.
